# The ichthyofauna of limnic systems in Quaternary deposits of extreme southern Brazil

**DOI:** 10.3897/zookeys.638.9199

**Published:** 2016-12-07

**Authors:** Cindy M. Assumpção, Fernando M. Quintela, Fabiano Corrêa, Daniel Loebmann

**Affiliations:** 1 Laboratorio de Vertebrados, Universidade Federal do Rio Grande, Instituto de Ciências Biológicas. Av. Itália km 8, Vila Carreiros, Rio Grande, Rio Grande do Sul, Brasil. CEP: 96203-900

**Keywords:** Biogeography, Cenozoic, coastal plain, endemism, fishes

## Abstract

The Quaternary in the state of Rio Grande do Sul (RS), southern Brazil, is geologically represented by the coastal plain and was originated by successive events of Pleistocene-Holocene marine transgressions and the occurrence of alluvial deposits. This paper aimed to characterize the fish assemblage occurring in a swampy Quaternary area adjacent to Lagoa Pequena, a lacustrine system connected to the west margin of the Laguna dos Patos estuary. A checklist is also provided of the ichthyofauna so far recorded in limnic systems of Quaternary deposits in the state of Rio Grande do Sul. A total of 42 species was recorded, distributed in nine orders, 18 families and 31 genera. Characidae and Cichlidae were the most representative families, comprising 15 and 4 species respectively. A bibliographic revision associated to our sample data revealed the occurrence of 156 species in limnic systems inserted in RS Quaternary deposits (114 limnic, 15 marine/estuarine/limnic, ten marine/estuarine, nine estuarine/limnic and eight marine). Characiformes and Siluriformes are the most diverse orders, corroborating the Neotropical pattern. Seven species can be considered endemic to RS Quaternary deposits.

## Introduction

The Quaternary in the state of Rio Grande do Sul (RS), southern Brazil, is geologically characterized by a sequence of four depositional events resulting from marine transgressions that occurred around 400,000 and 5,000 years ago, in addition to the occurrence of extensive alluvial systems (Tomazelli and Villwock 2000; Villwock and Tomazelli 2007). These Quaternary deposits are hydrographically heterogeneous and can be found in swamps, extensive floodplains (várzeas), coastal lagoons, coastal streams and lower stretches of fluvial systems originated from older geological formations in RS (Vieira 1984). Depositional events that occurred in RS during the Quaternary period also shaped the Patos-Mirim lagunar complex and resulted in the formation of the Patos Lagoon, the largest choked coastal lagoon worldwide (Kjerfve 1986; Möller and Fernandes 2010).

In relation to the ichthyofauna, limnic systems enclosed in RS Quaternary deposits home characteristically limnic, estuarine and coastal marine species, the last two due to temporary or permanent connections with estuarine and oceanic environments that allowed specimens to migrate (Tagliani 1994; Loebmann and Vieira 2005; Malabarba et al. 2013; Bastos et al. 2013). Sampling efforts on these limnic systems, especially on greater water bodies located in the coastal peninsular deposits (*restingas*), which include the Taim wetlands (Buckup and Malabarba 1983; Reis 1983; Grosser et al. 1994; Garcia et al. 2006), Lagoa Mangueira (Artioli et al. 2009), Lagoa do Peixe (Loebmann and Vieira 2005) and the northern complex of coastal lagoons (Schifino et al. 2004; Malabarba and Isaia 1992; Malabarba et al. 2013), provided consistent data on the species composition in these areas. However, information on fish assemblages present in limnic systems of Quaternary deposits located at the west margin of the Patos-Mirim complex is scarce. Data available on this segment is restricted to investigations by Becker et al. (2007) on the *Butiazais* region of the Tapes and the recent inventories produced by Volcan et al. (2012) and Burns et al. (2015) on the lower courses of the Corrientes and Turuçu streams, respectively. On such account, this study aimed to characterize the ichthyofauna in a swampy area adjacent to Lagoa Pequena, a lacustrine system under estuarine influence located at the west margin of the Patos Lagoon. We compare the assemblage found in the study area to other assemblages recorded in RS Quaternary deposits. A checklist is also presented of species recorded in limnic systems thus far in this geological formation, along with a brief discussion on distribution patterns of the species.

## Material and methods

### Study area

**Figure 1.**
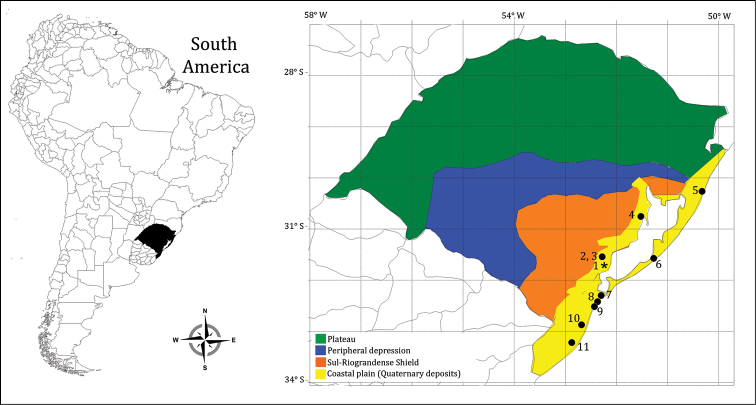
Four main geological formations from the state of Rio Grande do Sul, Southern Brazil. The study area (Quaternary deposits) as well as previous studies used in Dice Similarity Index analysis is entirely inserted in Coastal Plain. Key: **1** present study **2** Turuçu River basin; Burns et al. 2015 **3** Corrientes Stream; Volcan et al. 2012 **4** Tapes *Butiazais*; Becker et al. 2007 **5** Fortaleza lagoon; Schifino et al. 2004 **6** Lagoa do Peixe; Loebmann and Vieira 2005 **7** Peat forest; Quintela et al. 2007 **8** Rio Grande coastal streams; Tagliani 1995 **9** Cassino coastal streams; Bastos et al. 2013 **10** Taim wetland; Garcia et al. 2006 **11** Lagoa Mangueira; Artioli et al. 2009.

The west margin of the Patos Lagoon is characterized by the presence of Pleistocene-Holocene sedimentary deposits, with the predominance of silty-clayey sand (Tomazelli and Villwock 2000; CPRM 2007). The swamps we studied are located around Lagoa Pequena, a lacustrine system with an area of approximately 4,000 km^2^, in the boundaries of the Pelotas and Turuçu municipalities (Fig. 1). Lagoa Pequena is connected to the Patos Lagoon estuary on its west margin and is subject to estuarine physical-chemical and biological influence (Alves et al. 2009).

Fishes were sampled in four marginal swamps: swamp 1 (S1) -31°56'96"S, 52°11'78"W – emergent vegetation (*Juncus* sp. L.), with higher incidence of floating species (*Azolla* sp. Lam.) during warm periods; swamp 2 (S2) -31°56'90"S, 52°12'02"W – predominance of floating macrophytes (*Azolla* sp., *Pistia stratiotes* L., *Salvinia* sp. Ség.); swamp 3 (S3) -31°56'50"S, 52°13'10"W – margins sparsely covered by *Juncus* sp. and higher concentration of floating species (*Salvinia* sp.) during warm periods; swamp 4 (S4) -31°56'80"S, 52°13'82"W – predominance of *Nymphoides indica* (L.) Kuntze. Distances between the swamps and Lagoa Pequena are respectively 101 m, 395 m, 1,386 m and 2,229 m.

### Sampling

The ichthyofauna of the study area was sampled seasonally during the year of 2010 with the use of a 5 m long, 2 m high seine net with a 5 mm mesh between adjacent nodes. We applied an effort of three seines by seasonal sampling campaign in each swamp, totalizing an effort of 48 seines. Captured individuals were euthanized in clove oil solution, fixed in 10% formalin, preserved in 70% ethanol and housed in the Fish Reference Collection of the Instituto de Ciências Biológicas at the Universidade Federal do Rio Grande (CIFURG) (Appendix 1).

### Data analysis

Aiming to evaluate similarities on species composition between the study area assemblage and other fish assemblages recorded in RS Quaternary deposits (Tagliani 1995; Schifino et al. 2004; Loebmann and Vieira 2005; Garcia et al. 2006; Becker et al. 2007; Quintela et al. 2007; Artioli et al. 2009; Volcan et al. 2012 [partial: P12-P15]; Burns et al. 2015 [partial: potamon zone]), we calculated the Dice Similarity Index (DSI) based on a matrix of species presence/absence, using 1,000 *bootstrap* resampling. Obtained values were submitted to a cluster analysis based on the Unweighted Pair Group Method with Arithmetic Means (UPGMA) and similarity relationships were expressed in the form of a dendrogram. Grouping significance was tested through an Analysis of Similarity (ANOSIM). Analyses were performed in PAST version 1.79 (Hammer et al. 2001).

Finally, we compiled data regarding fish species recorded in limnic systems of RS Quaternary deposits from the list of the binary matrix used in the similarity analysis and from the literature, including information on additional species contained in punctual records, references on type material and references on comparative systematic/taxonomic studies (Malabarba and Isaia 1992; Buckup and Reis 1997; Costa and Cheffe 2001 2002 2005; Costa 2002 2006; Giora et al. 2008; Malabarba and Dyer 2002; Lucinda 2005 2008; Costa and Lanés 2009; Claudino et al. 2010; Volcan et al. 2010; Corrêa et al. 2011; Carvalho and Reis 2011; Malabarba et al. 2013; Lanés et al. 2015; Giora and Malabarba 2016).

## Results

A total of 4,206 individuals was captured in the four marginal-lacustrine swamps sampled. They were distributed in nine orders, 18 families, 31 genera and 42 species. Characidae and Cichlidae were the most representative families, comprising 15 and four species respectively. All other families were represented by two or one single species (Table 1).

**Table 1.** Checklist of fish species recorded in limnic systems of Quaternary deposits in Rio Grande do Sul State. References: 1) Malabarba and Isaia (1992), 2) Tagliani (1994), 3) Buckup and Reis (1997), 4) Costa and Cheffe (2001), 5) Costa (2002), 6) Costa and Cheffe (2002), 7) Malabarba and Dyer (2002), 8) Schifino et al. (2004), 9) Costa and Cheffe (2005), 10) Loebmann and Vieira (2005), 11) Lucinda (2005), 12) (Costa (2006), 13) Garcia et al. (2006), 14) Becker et al. (2007), 15) Quintela et al. (2007), 16) Giora et al. (2008), 17) Lucinda (2008), 18) Artioli et al. (2009), 19) Costa and Lanés (2009), 20) Claudino et al. (2010), 21) Volcan et al. (2010), 22) Correa et al. (2011), 23) Carvalho and Reis (2011), 24) Volcan et al. (2012), 25) Bastos et al. (2013), 26) Malabarba et al. (2013), 27) Lanés et al. (2015), 28) Burns et al. (2015), 29) Giora and Malabarba (2016); PS = present study, M (marine), E (estuarine), L (limnic).

Taxon			Habit	References
Clupeiformes				
	Clupeidae			
		*Brevoortia pectinata* (Jenyns, 1842)	M,E	10, 28
		*Harengula clupeola* (Cuvier, 1829)	M	10
		*Platanichthys platana* (Regan,1917)	E, L	8, 10, 13, 14, 18, 25, 26, 28, PS
		*Sardinella aurita* Valenciennes, 1847	M, E	10
Elopiformes				
	Elopidae			
		*Elops saurus* Linnaeus, 1766	M, E	10
Albuliformes				
	Albulidae			
		*Albula nemoptera* Fowler, 1911	E,L	10
Gadiformes				
	Phycidae			
		*Urophycis brasiliensis* (Kaup, 1858)	M	10
Mugiliformes				
	Mugilidae			
		*Mugil curema* Valenciennes, 1836	M,E,L	10, 25
		*Mugil brevirostris* (Ribeiro, 1915)	M,E,L	10, 25
		*Mugil liza* Valenciennes, 1836	M,E,L	10, 25, 28, PS
	Engraulidae			
		*Anchoa marinii* Hildebrand, 1943	M	10
		*Lycengraulis grossidens* (Agassiz, 1829)	M,E	8, 10, 24, 25, PS
Characiformes				
Acestrorhynchidae				
		*Acestrorhynchus pantaneiro* Menezes, 1992	L	26
	Characidae			
		*Aphyocharax anisitsi* Eigenmann & Kennedy, 1903	L	13, 14, 18, 24, 26
		Astyanax aff. fasciatus (Cuvier, 1819)	L	1, 8, 10, 13, 14, 15, 18, 24, 26, PS
		*Astyanax eigenmanniorum* (Cope, 1894)	L	1, 2, 8, 10, 13, 14, 15, 18, 24, 25, 26, 28, PS
		*Astyanax henseli* Melo & Buckup, 2006	L	21, PS
		*Astyanax lacustris* (Lütken, 1875)	L	1, 8, 10, 13, 14, 18, 25, 28, PS
		*Astyanax laticeps* (Cope, 1894)	L	21
		*Astyanax stenohalinus* Messner, 1962	L	28
		*Bryconamericus iheringii* (Boulenger, 1887)	L	13, 14, 18, 28
		*Charax stenopterus* (Cope, 1894)	L	1, 8, 13, 14, 18, 24, 26, 28, PS
		*Cheirodon ibicuhiensis* Eigenmann, 1915	L	1, 10, 13, 14, 15, 18, 24, 25, 26, 28, PS
		*Cheirodon interruptus* (Jenyns, 1842)	L	1, 2, 10, 14, 15, 18, 24, 25, 26, 28, PS
		*Diapoma alburnus* (Hensel, 1870)	L	1, 10, 13, 14, 15, 18, 24, 26, 28, PS
		*Diapoma speculiferum* Cope, 1894	L	24, 28
		*Hyphessobrycon boulengeri* (Eigenmann, 1907)	L	1, 2, 10, 13, 14, 15, 24, 25, 26, 28, PS
		*Hyphessobrycon igneus* Miquelarena, Menni, López & Casciotta, 1980	L	1, 2, 10, 13, 14, 18, 24, 25, 26, 28, PS
		*Hyphessobrycon luetkenii* (Boulenger, 1887)	L	2, 8, 10, 13, 14, 15, 18, 24, 25, 26, 28, PS
		*Hyphessobrycon meridionalis* Ringuelet, Miquelarena & Menni, 1978	L	1, 2, 10, 13, 14, 18, 24, 25, 26, 28, PS
		*Hyphessobrycon togoi* Miquelarena & López, 2006	L	26, PS
		*Macropsobrycon uruguayanae* Eigenmann, 1915	L	13, 18, 28
		*Mimagoniates inequalis* (Eigenmann, 1911)	L	10, 24, 25, 26, 28
		*Mimagoniates microlepis* (Steindachner, 1877)	L	1, 26
		*Oligosarcus jenynsii* (Günther, 1864)	L	1, 2, 8, 10, 13, 14, 15, 18, 24, 25, 26, 28, PS
		*Oligosarcus robustus* Menezes, 1969	L	1, 2, 8, 10, 13, 14, 18, 25, 26, 28, PS
		*Pseudocorynopoma doriae* Perugia, 1891	L	1, 10, 13, 14, 24, 26, 28, PS
		*Serrapinnus calliurus* (Boulenger, 1900)	L	14
	Crenuchidae			
		*Characidium aff. zebra* Eigenmann, 1909	L	14, 26
		*Characidium orientale* Buckup & Reis, 1997	L	3, 24, 23, 28, PS
		*Characidium rachovii* (Regan, 1913)	L	3, 10, 13, 14, 18, 23, 24, 28, PS
		*Characidium pterostictum* Gomes, 1947	L	28
		*Characidium tenue* (Cope, 1894)	L	14, 18, 28
	Curimatidae			
		*Cyphocharax saladensis* (Meinken, 1933)	L	1, 10, 14, 24, 26, 28
		*Cyphocharax voga* (Hensel, 1870)	L	1, 2, 8, 10, 13, 14, 18, 24, 26, 28, PS
		*Steindachnerina biornata* (Braga & Azpelicueta, 1987)	L	1, 24, 26, 28, PS
	Erythrinidae			
		*Hoplias malabaricus* (Bloch, 1794)	L	1, 2, 8, 10, 13, 14, 18, 24, 25, 26, 28, PS
	Lebiasinidae			
		*Pyrrhulina australis* (Eigenmann & Kennedy, 1903)	L	1, 14, 26
Siluriformes				
	Ariidae			
		*Genidens genidens* (Cuvier, 1829)	M,E	10, 28
	Aspredinidae			
		*Bunocephalus erondinae* Cardoso, 2010	L	28
		*Pseudobunocephalus iheringii* (Boulenger, 1891)	L	13, 14, 28
	Auchenipteridae			
		Glanidium cf. catharinensis Miranda Ribeiro, 1962	L	26
		*Trachelyopterus lucenai* Bertoletti, Silva & Pereira, 1995	L	8, 13, 14, 18, 24, 26
	Callichthyidae			
		*Callichthys callichthys* (Linnaeus, 1758)	L	1, 10, 13, 24, 25, 26, 28
		*Corydoras paleatus* (Jenyns, 1842)	L	1, 2, 8, 10, 13, 14, 15, 18, 24, 25, 26, 28, PS
		*Corydoras undulatus* (Regan, 1912)	L	1,26
		*Hoplosternum littorale* (Hancock, 1828)	L	1, 10, 13, 14, 18, 24, 25, 26, 28, PS
		*Lepthoplosternum tordilho* Reis, 1997	L	14
	Heptapteridae			
		*Heptapterus sympterygium* Buckup, 1988	L	1, 2, 13, 24, 25, 26, 28
		*Heptapterus mustelinus* (Valenciennes, 1835)	L	28
		*Rhamdella* sp.	L	26
		*Pimelodella australis* Eigenmann, 1917	L	1, 2, 10, 13, 14, 18, 24, 26, PS
		*Rhamdella eriarcha* (Eigenmann & Eigenmann, 1888)	L	1, 28
		Rhamdia aff. quelen (Quoy & Gaimard, 1824)	L	1, 2, 8, 10, 13, 14, 15, 18, 24, 25, 26, 28, PS
	Loricariidae			
		*Ancistrus brevipinnis* (Regan, 1904)	L	14, 28
		*Hisonotus laevior* Cope, 1894	L	28, PS
		*Hisonotus leucofrenatus* (Ribeiro, 1908)	L	26
		*Hisonotus nigricauda* (Boulenger, 1891)	L	23, 24, 28
		*Hisonotus armatus* Carvalho, Lehmann, Pereira & Reis, 2008	L	28
		*Hisonotus taimensis* (Buckup, 1981)	L	2, 13, 18, 24
		*Hypostomus aspilogaster* (Cope, 1894)	L	28
		*Hypostomus commersoni* (Valenciennes, 1836)	L	1, 13, 14, 18, 26, 28
		*Loricariichthys anus* (Valenciennes, 1836)	L	1, 8, 13, 14, 18, 24, 26, 28
		*Otothyris rostrata* (Garavello, Britski & Schaefer, 1998)	L	26, 28
		*Otocinclus flexilis* Cope, 1894	L	28
		*Rineloricaria cadeae* (Hensel, 1868)	L	13, 14, 18, 24, 28
		*Rineloricaria longicauda* Reis, 1983	L	1, 13, 18, 28
		*Rineloricaria quadrensis* Reis, 1983	L	1, 8, 26
		*Rineloricaria microlepidogaster* (Regan, 1904)	L	28
		*Rineloricaria strigilata* (Hensel, 1868)	L	14,18, 28
	Pimelodidae			
		*Parapimelodus nigribarbis* (Boulenger, 1889)	L	13, 18, 28
		*Pimelodus pintado* Azpelicueta, Lundberg & Loureiro, 2008	L	13, 14, 18, 28
	Pseudopimelodidae			
		*Microglanis cibelae* Malabarba & Mahler, 1998	L	26
		*Microglanis cottoides* (Boulenger, 1891)	L	2, 13, 14, 18, 28
	Trichomycteridae			
		*Scleronema* sp. aff. *Scleronema operculatum* Eigenmann, 1917	L	28
		*Homodiaetus anisitsi* Eigenmann & Ward, 1907	L	1, 13, 14, 18, 24, 28
Gymnotiformes				
	Gymnotidae			
		*Gymnotus omarorum* Richer-de-Forges, Crampton & Albert, 2009	L	28
		*Gymnotus refugio* Giora & Malabarba, 2016	L	29
		Gymnotus aff. carapo Linnaeus, 1758	L	1, 13,14
	Hypopomidae			
		*Brachyhypopomus bombilla* Loureiro & Ana Silva, 2006	L	24, 28
		*Brachyhypopomus draco* Giora, Malabarba & Crampton, 2008	L	16, 20, 26, PS
		*Brachyhypopomus gauderio* Giora & Malabarba, 2009	L	22, 24, 26, 28, PS
	Sternopygidae			
		*Eigenmannia trilineata* López & Castello, 1966	L	1, 2, 10, 13, 14, 24, 26, 28
Cyprinodontiformes				
	Anablepidae			
		*Jenynsia multidentata* (Jenyns, 1842)	E,L	2, 10, 13, 14, 15, 18, 24, 25, 26, 28, PS
	Poeciliidae			
		*Cnesterodon decemmaculatus* (Jenyns, 1842)	E,L	2, 10, 13, 15, 18, 26, 28
		*Phalloceros caudimaculatus* (Hensel, 1868)	E,L	1, 2, 13, 14, 15, 18,24, 25, 26, 28, PS
		*Phalloceros spiloura* Lucinda, 2008	L	17
		*Phalloptychus iheringi* (Boulenger, 1889)	L	10, 11, 26, PS
		*Poecilia vivipara* Bloch & Schneider, 1801	E,L	1, 26
	Cynolebiidae			
		*Atlantirivulus riograndensis* (Costa & Lanés, 2009)	L	19, 26
		*Austrolebias adloffi* (Ahl,1922)	L	12, 14
		*Austrolebias charrua* Costa & Cheffe, 2001	L	4, 12, 21
		*Austrolebias jaegari* Costa & Cheffe, 2002	L	6, 12
		*Austrolebias luteoflammulatus* (Vaz-Ferreira, Sierra de Soriano & Scaglia de Paulete, 1965)	L	12, 21
		*Austrolebias minuano* Costa & Cheffe, 2001	L	4, 12, 15, 24
		*Austrolebias natchtigalli* Costa, 2006	L	12
		*Austrolebias nigrofasciatus* Costa & Cheffe, 2001	L	4, 12
		*Austrolebias prognathus* (Amato, 1986)	L	21
		*Austrolebias univentripinnis* Costa & Cheffe, 2005	L	9
		*Austrolebias wolterstorffi* (Ahl, 1924)	L	12, 15, 27
		*Cynopoecilus fulgens* Costa, 2002	L	5, 27
		*Cynopoecilus melanotaenia* (Regan, 1912)	L	2, 5, 13, 15, 21, 28, PS
		*Cynopoecilus multipapillatus* Costa, 2002	L	5, 27
		*Cynopoecilus nigrovittatus* Costa, 2002	L	14
Atheriniformes				
	Atherinopsidae			
		*Atherinella brasiliensis* (Quoy & Gaimard, 1825)	M,E	10, 24, 26, PS
		Odontesthes aff. perugiae Evermann & Kendall, 1906	L	13, 18
		*Odontesthes argentinensis* (Valenciennes, 1835)	M,E,L	10, 26, PS
		*Odontesthes bicudo* Malabarba & Dyer, 2002	L	7, 26
		*Odontesthes bonariensis* (Valenciennes, 1835)	M,E,L	13, 18, 26
		*Odontesthes humensis* de Buen, 1953	L	13, 18
		*Odontesthes ledae* Malabarba & Dyer, 2002	E,L	7, 8, 26
		*Odontesthes mirinensis* Bemvenuti, 1995	L	13, 18
		*Odontesthes piquava* Malabarba & Dyer, 2002	L	7, 26
		*Odontesthes retropinnis* de Buen, 1953	L	18
Perciformes				
	Carangidae			
		*Selene vomer* (Linnaeus, 1758)	M,E	10
		*Trachinotus carolinus* (Linnaeus, 1766)	M,E	10
		*Trachinotus marginatus* Cuvier, 1832	M	10, 25
		*Uraspis secunda* (Poey, 1860)	M	10
	Centropomidae			
		*Centropomus parallelus* Poey, 1860	M,E,L	10
	Gerreidae			
		*Eucinostomus argenteus* Baird & Girard, 1855	M,E,L	10
		*Eucinostomus melanopterus* (Bleeker, 1863)	M,E,L	25
	Lutjanidae			
		*Lutjanus cyanopterus* (Cuvier, 1828)	M,E	25
	Pomatomidae			
		*Pomatomus saltatrix* Linnaeus, 1776	M,E	10
	Sciaenidae			
		*Micropogonias furnieri* (Desmarest, 1823)	M,E	10, 25
		*Pachyurus bonariensis* Steindachner, 1879	L	14
		*Pogonias cromis* Linnaeus, 1766	M,E	10
		*Stellifer brasiliensis* (Schultz, 1945)	M	10
	Epinephelidae			
		*Epinephelus marginatus* Lowe, 1834	M	10
		*Mycteroperca acutirostris* (Velenciennes, 1828)	M	10
Labriformes				
	Cichlidae			
		*Australoheros acaroides* (Hensel, 1870)	L	1, 2, 10, 13, 14, 18, 24, 25, 28, PS
		*Cichlasoma portalegrense* (Hensel, 1870)	L	10, 13, 14, 18, 24, 25, 26, 28, PS
		*Crenicichla lepidota* Heckel, 1840	L	1, 2, 8, 10, 13, 14, 18, 24, 25, 26, 28, PS
		*Crenicichla maculata* Kullander & Lucena, 2006	L	26
		*Crenicichla punctata* Hensel, 1870	L	8, 13, 18, 28
		*Geophagus brasiliensis* (Quoy & Gaimard, 1824)	L	1, 2, 8, 10, 13, 14, 18, 24, 25, 26, 28, PS
		*Gymnogeophagus gymnogenys* (Hensel, 1870)	L	1, 13, 14, 18, 26, 28
		*Gymnogeophagus lacustris* Reis & Malabarba, 1988	L	1, 26
		*Gymnogeophagus rhabdotus* (Hensel, 1870)	L	1, 13, 14, 18, 24, 26, 28
Gobiiformes				
	Eleotridae			
		*Dormitator maculatus* (Bloch, 1792)	M,E,L	10, 25
		*Eleotris pisonis* (Gmelin, 1789)	M,E,L	10, 25
	Gobiidae			
		*Awaous tajasica* (Lichtenstein, 1822)	E,L	10
		*Ctenogobius shufeldti* (Jordan & Eigenmann, 1887)	E,L	10, 13, 18, 24, 25, 26, PS
		*Gobionellus oceanicus* (Pallas, 1770)	M,E,L	10
Pleuronectiformes				
	Paralichthyidae			
		*Citharichthys spilopterus* Günther, 1862	M,E,L	10
		*Paralichthys orbignyanus* (Valenciennes, 1839)	M,E	10, 24
Synbranchiformes				
	Synbranchidae			
		*Synbranchus marmoratus* Bloch, 1795	L	1, 2, 13, 14, 15, 24, 25, 26, 28, PS

DSI values (Table 2) showed that the assemblage found in marginal swamps is most similar to the assemblages found in the lower course of the Corrientes Stream (Volcan et al. 2012) and the Lagoa do Peixe National Park (DSI = 0.674; Table 2), while the most dissimilar assemblages included a set of three coastal streams sampled by Tagliani (1995) in the Rio Grande municipality (DSI = 0.394; Table 2). Values for other comparisons are shown in Table 2.

**Table 2.** Values of Dice Similarity Index (DSI) between ichthyocenoses recorded in limnic systems of Rio Grande do Sul Quaternary deposits. Legend: AC (Rio Grande coastal streams; Tagliani [1995]), BU (Tapes *Butiazais*; Becker et al. [2007]), CO (Corrientes Stream; Volcan et al. [2012]), LDP (Lagoa do Peixe; Loebmann and Vieira [2005]), LF (Lagoa Fortaleza; Schifino et al. [2004]), LM (Lagoa Mangueira; Artioli et al. [2009]), LP (Lagoa Pequena marginal marshes; present study), MP (peat forest; Quintela et al. [2007]), SA (Cassino coastal streams; Bastos et al. [2013]), TA (Taim wetland; Garcia et al. [2006]), TU (Turuçu River basin; Burns et al. [2015]).

	BU	TA	LDP	SA	AC	LM	LP	MP	LF	CO	TU
BU	1	0.745	0.524	0.484	0.390	0.729	0.586	0.432	0.421	0.641	0.574
TA	0.745	1	0.544	0.547	0.463	0.897	0.566	0.405	0.447	0.660	0.620
LDP	0.524	0.544	1	0.705	0.427	0.52	0.674	0.388	0.406	0.542	0.475
AS	0.484	0.547	0.705	1	0.418	0.522	0.548	0.475	0.328	0.477	0.368
AC	0.390	0.463	0.427	0.418	1	0.405	0.394	0.435	0.375	0.373	0.297
LM	0.729	0.897	0.520	0.522	0.405	1	0.542	0.394	0.466	0.580	0.571
LP	0.586	0.566	0.674	0.548	0.394	0.542	1	0.444	0.462	0.674	0.542
MP	0.432	0.405	0.388	0.475	0.435	0.394	0.444	1	0.350	0.328	0.258
LF	0.421	0.447	0.406	0.328	0.375	0.466	0.462	0.350	1	0.348	0.316
CO	0.641	0.660	0.542	0.477	0.373	0.580	0.674	0.328	0.348	1	0.639
TU	0.574	0.620	0.475	0.368	0.297	0.571	0.542	0.258	0.316	0.639	1

**Figure 2.**
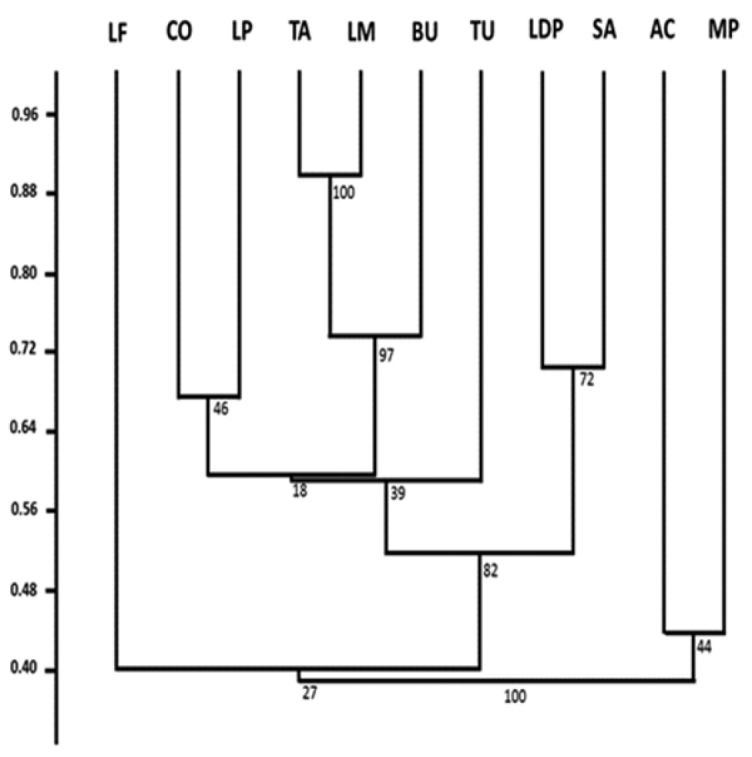
Dendrogram generated from the values of DSI between ichthyocenoses recorded in limnic systems of Rio Grande do Sul Quaternary deposits. Key: AC (Rio Grande coastal streams; Tagliani [1995]), BU (Tapes *Butiazais*; Becker et al. [2007]), CO (Corrientes Stream; Volcan et al. [2012]), LDP (Lagoa do Peixe; Loebmann and Vieira [2005]), LF (Lagoa Fortaleza; Schifino et al. [2004]), LM (Lagoa Mangueira; Artioli et al. [2009]), LP (Lagoa Pequena marginal marshes; present study), MP (Peat forest; Quintela et al. [2007]), SA (Cassino coastal streams; Bastos et al. [2013]), TA (Taim wetland; Garcia et al. [2006]), TU (Turuçu River basin; Burns et al. [2015]).

The dendrogram obtained from DSI values (Fig. 2) showed the formation of four clusters. The cluster with higher support (*bootstrap* = 97) gathers the assemblages of Taim (Garcia et al. 2006), Lagoa Mangueira (Artioli et al. 2009) and the *Butiazais* region of Tapes (Becker et al. 2007). Another cluster (*bootstrap* = 72) was formed by the assemblages of Lagoa do Peixe (Loebmann and Vieira 2005) and three coastal streams sampled by Bastos et al. (2013) in Cassino beach. The assemblage from our study area clustered with the ichthyocenose from the Corrientes stream’s lower course (Volcan et al. 2012), with low support (*bootstrap* = 46). The assemblages of a peat forest fragment (Quintela et al. 2007) and a set of coastal streams in Rio Grande (Tagliani 1995) grouped with low support (*bootstrap* = 44). The assemblages of Lagoa Fortaleza (Schifino et al. 2004) and Turuçu River (Burns et al. 2015) remained isolated. The ANOSIM indicated significant differences between the clusters (*p* = 0.0003; R = 0.98).

## Discussion

### Marginal-lacustrine swamps

The marginal-lacustrine swamps sampled in the present study host a considerable ichthyofaunistic diversity, showing a species richness within the range observed in limnic systems of RS Quaternary deposits. For example, Garcia et al. (2006) recorded 57 species in lagoons of the Taim Ecological Station in the Rio Grande *restinga*, while Artioli et al. (2009) sampled 52 species in Lagoa Mangueira. Smaller systems in this *restinga* have shown lower richness, such as 31 species in a set of three coastal streams (Tagliani 1995) and 18 species in a peat forest fragment (Quintela et al. 2007). Loebmann and Vieira (2005) recorded 67 species (among limnic, estuarine and marine forms) in Lagoa do Peixe at the *restinga* of São José do Norte, while Schifino et al. (2004) listed 22 species in Lagoa Fortaleza, at the Tramandaí River basin. Becker et al. (2007) recorded 55 species in alluvial deposits of swamps, ponds and streams on the west margin of the Patos-Mirim complex, at the *Butiazais* region in Tapes, while Volcan et al. (2012) found 49 species in the Corrientes Stream’s lower course. Such a high species richness registered for characids, which represent 34% of the species recorded in the study area, corroborates with the pattern found in other fish assemblages previously investigated in RS Quaternary deposits (e.g. Tagliani 1995; Schifino et al. 2004; Loebmann and Vieira 2005; Garcia et al. 2006; Becker et al. 2007; Quintela et al. 2007; Artioli et al. 2009; Volcan et al. 2012; Bastos et al. 2013).

Regarding system comparisons, the assemblage of marginal-lacustrine swamps is more similar on its species composition to the assemblage of the lower course of the Corrientes Stream (Volcan et al. 2012), followed by the ichthyocenose of the Lagoa do Peixe National Park (Loebmann and Vieira 2005). The Corrientes Stream, however, is closest to the study area when compared to the other systems, distancing from 50 m up to 590 m from the marginal swamps. Thirty-four of the forty-two species (81%) recorded in the marginal swamps were also found by Volcan et al. (2012) in the Corrientes Stream’s lower course and the following species were found exclusively in the swamps: *Odontesthes argentinensis*, *Platanichthys platana*, *Mugil liza*, *Astyanax* aff. *fasciatus*, *Hyphessobrycon togoi*, *Brachyhypopomus draco*, *Hisonotus laevior* and *Phalloptychus iheringi*. Such similarity may be related to the proximity and possible connection between the stream and marshes during rainfall periods, leading to an exchange of species. The second most similar assemblage of the Lagoa do Peixe National Park (Loebmann and Vieira 2005), on the other hand, is located in deposits at the eastern margin of the Patos Lagoon. The fact that higher similarity is found between assemblages from the study area and Lagoa do Peixe is remarkable, considering that other systems such as the Turuçu basin (Burns et al. 2015) and the systems of Rio Grande (Tagliani et al. 1995; Quintela et al. 2007; Bastos et al. 2013), are closer to the study area. These results suggest that environmental factors may exert greater influence on the species composition in these systems compared to the effect of geographic distances.

With the exception of *Characidium orientale*, all other species found in the study area have been recorded in limnic systems of the coastal *restingas* of Rio Grande and São José do Norte (Malabarba and Isaia 1992; Tagliani 1995; Loebmann and Vieira 2005; Garcia et al. 2006; Quintela et al. 2007; Artioli et al. 2009; Bastos et al. 2013; Malabarba et al. 2013). *Characidium orientale* was described among specimens collected in Arroio Chasqueiro and the type series includes specimens from several localities in the RS Shield (Precambrian) and the Central Depression (Permian-Triassic) (Buckup and Reis 1997). Posteriorly, the species was recorded by Volcan et al. (2012) in the Corrientes Stream and by Burns et al. (2015) in the Turuçu River basin. Therefore, this species’ distribution in the RS Quaternary may be restricted to alluvial deposits of the west margin of the Patos-Mirim complex.

### Ichthyofauna of limnic systems in the Quaternary deposits of Rio Grande do Sul

The compilation of bibliographic information associated to our sample data revealed a total of 156 species (114 limnic, 15 marine/estuarine/limnic, ten marine/estuarine, nine estuarine/limnic and eight marine) occurring in limnic systems inserted in RS Quaternary deposits (Table 1). Comparatively, 160 species (including undescribed forms) can be found in the Patos Lagoon basin (Malabarba et al. 2009), which includes rivers and streams originated from distinct geological formations in RS such as the Shield and the Paraná Basin (Central Depression and Meridional Plateau), located beyond the Patos Lagoon itself, which in turn, covers an area of approximately 10,360 km^2^ (Vieira 1984). Approximately 100 species were recorded in the Tramandaí River basin, which is formed by rivers and streams of the Serra Geral slops (eastern Meridional Plateau) and several lakes and channels of the northern coastal plain (Malabarba et al. 2013). Therefore, limnic systems in the RS Quaternary host relevant ichthyofaunistic diversity, compassing approximately 27% of the freshwater ichthyofauna recorded in RS (Bertaco et al. 2016). Furthermore, this study recorded a low percentage (around 8%) of the marine species usually found in the RS coastal zone (Seeliger et al. 1998).

The Siluriformes and Characiformes orders, which comprise respectively 38 and 36 species, were the most speciose orders in RS Quaternary deposits, corroborating with the pattern already found for the Neotropical region (Lowe-McConnell 1987; Reis et al. 2003a). With the exception of *Acestrorhyncus pantaneiro*, a species typical for the Paraná, Uruguay, Paraguay and Mamoré River basins (Menezes 2003), and *Pachyurus bonariensis*, native from the Paraná, Uruguay and Paraguay River basins (Casatti 2003), all other recorded species are characteristic of Atlantic basins in RS, which include the Patos-Mirim basin and Tramandaí and Mampituba rivers (Buckup and Reis 1997; Melo and Buckup 2006; Malabarba 2008; Malabarba et al. 2013). *Acestrorhyncus pantaneiro* was recorded for the first time in RS Atlantic basins by Saccol-Pereira et al. (2006), who reported the capture of three individuals in the Parque Estadual Delta do Jacuí during the years of 2004 and 2005. Posteriorly, Artioli et al. (2013) reported the capture of three more individuals during the year of 2008 in lagoons in Fortaleza and Malvas and in the Tramandaí basin. Recently, Einhardt et al. (2014) registered the species in the Chasqueiro Stream micro-basin, an integrant of the Mirim Lagoon sub-basin. Before these records, the occurrence of *Acestrorhyncus pantaneiro* in RS was known only for the Uruguay River basin, where it is considered a native species (Menezes 2003). Thus, this phenomenon represents a case of recent invasion and dispersion of an aloctone species in RS Atlantic basins, which according to Artioli et al. (2013), could have been favored by the geomorphology of the coastal plain, associated to connectivity between systems during flooding periods and the opening of artificial channels used for drainage and irrigation.

An analysis of the geographic distribution of species occurring in RS Quaternary deposits reveals distinct patterns. One group of “subtropical” species is distributed mainly in the Pampa biome, although some species also spread out to peripheral systems of subtropical Atlantic Forest and to other contacting biomes (e.g. *Astyanax eigenmanniorum*, *Cheirodon interruptus*, *Hyphessobrycon meridionalis*, *Hyphessobrycon igneus*, *Hyphessobrycon togoi*, *Oligosarcus jenynsii*, *Cyphocarax voga*, *Loricariichthys anus*, *Odontesthes bonariensis*, *Phalloceros caudimaculatus*). These species are typical for the Tramandaí, Patos-Mirim, Uruguay and lower Paraná basins (Malabarba 1998; Dyer 2003; Ferraris 2003; Lima et al. 2003; Lucinda 2008; Malabarba et al. 2013). Most of them are widely distributed in the RS Quaternary, occurring in systems from all segments of the coastal *restingas* and also in alluvial plains at west of the Patos-Mirim complex (Loebmann and Vieira 2005; Artioli et al. 2009; Volcan et al. 2012; Malabarba et al. 2013; present study). One sub-group of “subtropical” species (e.g. *Australoheros acaroides*, *Cichasoma portalegrense*, *Gymnogeophagus gymnogenys*, *Oligosarcus robustus*, *Heptapterus sympterygium*) is restricted to Atlantic discharge basins in RS (Reis and Malabarba 1988; Schindler et al. 2010; Malabarba et al. 2013) and is also widespread in RS Quaternary deposits (Artioli et al. 2009; Bastos et al. 2013; Malabarba et al. 2013; as well as the present study). Meanwhile, another sub-group is restricted to specific segments of the RS Quaternary deposits. For example, *Rineloricaria quadrensis*, *Microglanis cibelae* and *Pachyurus bonariensis*, natives from the Paraná, Uruguay and Paraguay basins (Casatti 2003), occur exclusively in lagoons and channels of the northern coastal plain and rivers of the Tramandaí basin (Malabarba et al. 2013); the second also occurring in the Mampituba basin (Malabarba and Mahler 1998).

The RS Quaternary deposits are also marked by endemism. *Odontesthes ledae*, *Odontesthes piquava*, *Odontesthes bicudo*, *Gymnogeophagus lacustris* and *Gymnotus refugio* occur only in systems of the Tramandaí basin (Reis and Malabarba 1988; Malabarba and Dyer 2002) and consequently represent endemic species of the northern coastal plain. Two other cases of endemism in the central and southern segments of coastal *restingas* are known: *Cynopoecilus fulgens* is known only in its typical locality (municipality of São José do Norte) (Costa 2002) and in the Lagoa do Peixe National Park (municipalities of Tavares and Mostardas) (Keppeler et al. 2015; Lanés et al. 2014 2015), therefore occupying a restrict portion of the central coastal plain; *Austrolebias minuano* was described among specimens collected in the Rio Grande municipality (Costa and Cheffe 2001). Posteriorly, this same species was recorded in new areas of Rio Grande (Porciuncula et al. 2006), in a *restinga* of São José do Norte, in the municipalities of Tavares and São José do Norte (Costa 2006), and in the Lagoa do Peixe National Park (Corrêa et al. 2009; Keppeler et al. 2015; Lanés et al. 2014, 2015).

In contrast, there is one group of species that is widely distributed in Brazil and in the Neotropical region and is also well distributed in the RS Quaternary, occurring both in coastal *restingas* and alluvial plains at the Patos-Mirim west margin. The callichthyids *Hoplosternum littorale* and *Callichthys callichthys* occur in a great portion of the cis-Adean South America (Reis 2003). The gobiid *Ctenogobius shufeldti* is spread from North Carolina (EUA) to southern Brazil (Malabarba et al. 2013). Among cichlids, *Crenicichla lepidota* occurs from the Guaporé River (Amazon basin) up to the Uruguay and Paraná basins, while *Geophagus brasiliensis* is distributed along eastern Brazilian coastal basins and Uruguayan systems (Kullander 2003). Other species that inhabit coastal basins of southeastern and southern Brazil are the characid *Hyphessobrycon boulengeri* and the anablepid *Jenynsia multidentata*, both also occurring in Uruguay and Argentina (Malabarba et al. 2013). However, some widespread taxa are currently recognized as a species complex, including *Astyanax* “*fasciatus*”, *Characidium* “*zebra*”, *Hoplias* “*malabaricus*”, *Gymnotus* “*carapo*”, *Rhamdia* “*quelen*” and *Synbranchus* “*marmoratus*” (Malabarba et al. 2013). Therefore, these forms lack an integrative systematic analysis and appropriate taxonomic definitions.

Biogeographic hypotheses on South American ichthyofauna date back to the early twentieth century (Ribeiro et al. 2013) and point to sea level oscillations and orogenetic effects as the main factors that shape distribution patterns (Malabarba and Isaia 1992; Ribeiro 2006; Ribeiro et al. 2013). The fact that few species from genera that are usually well represented in “inland basins” occur in condition of endemism in the coastal basin (e.g. *Crenicichla* and *Gymnogeophagus*) suggests the occurrence of vicariance events followed by cladogenesis. Indeed, speciation by vicariance involving the genera *Gymnogeophagus* (Reis and Malabarba 1988; Malabarba and Isaia 1992), *Mimagoniates* (Menezes and Weitzman 1990) and *Odontesthes* (Malabarba and Dyer 2002) is suggested as one of the evolutionary processes that occurred in the Tramandaí River basin. Molecular data (Beheregaray et al. 2001) revealed that three endemic *Odontesthes* species in the Tramandaí basin (*Odontesthes bicudo*, *Odontesthes ledae* and *Odontesthes piquava*) have probably diverged after the Pleistocene-Holocene marine regressions that shaped the complex of lagoons where these species occur almost allopatrically. The “Pattern C” proposed by Ribeiro (2006), which suggests the occurrence of recent intraspecific vicariance events between “inland basins of the Brazilian Shield” and “coastal basins”, is corroborated by the presence of species whose distribution is restricted to the Atlantic drainage basins in RS (Patos and Tramandaí) and the “adjacent inland basin” of the Uruguay River - those species being *Astyanax* aff. *fasciatus*, *Gymnogeophagus rhabdotus* and *Mimagoniates inequalis*. Therefore, the extant ichthyofaunistic composition of limnic systems in RS Quaternary deposits seems to be the result of both internal processes and evolutionary events triggered in older adjacent geological formations.

In conclusion, limnic systems of Quaternary RS deposits host a diversified ichthyofauna, including endemic species and species with restricted distribution (to the RS state). These systems are home to 15 endangered species at state level (State Decree 51.797/2014), which include 13 killifishes (Rivulidae), *Odonthestes bicudo* and *Gymnotus refugio*. Rivulids, as well as other representatives of the ichthyofauna, are affected by the destruction and alteration of aquatic environments. In RS, interferences caused by rice cultivation, livestock, silviculture and urbanization are the main threats to the freshwater ichthyofauna (Reis et al. 2003b; Volcan et al. 2010). Moreover, only two integral protection conservation units host populations of endangered rivulids along the entire domain of the RS Quaternary deposits: the Banhado do Maçarico Biological Reserve (Costa 2006) and the Lagoa do Peixe National Park (Corrêa et al. 2009; Lanés et al. 2015). Thus, most of the threatened killifish populations, as well as populations of *Odonthestes bicudo* and *Gymnotus refugio*, remain unprotected. In this context, conserving these and other species, as pointed out by Reis et al. (2003b) and Volcan et al. (2012), implies the creation of public and private conservation units.

## Appendix 1

List of vouchers housed in the Fish Reference Collection of FURG (CIFURG), Instituto de Ciências Biológicas, Universidade Federal do Rio Grande, Rio Grande do Sul, Brazil.

*Astyanax eigenmanniorum*: CIFURG 22, 24, 39, 51, 65, 82, 101, 107, 113, 117, 126, 148, 159, 169, 179, 196; *Astyanax fasciatus*: CIFURG 23, 96, 125, 197; *Astyanax henseli*: CIFURG 25, 62, 67, 173, 180; *Astyanax lacustris*: CIFURG 50, 70, 90, 146, 162, 167, 175; *Atherinella brasiliensis*: CIFURG 78, 109; *Australoheros acaroides*: CIFURG 35, 58, 85, 99, 145, 153, 191; *Brachyhypopomus draco*: CIFURG 57, 123; *Brachyhypopomus gauderio*: CIFURG 15, 110, 124, 143; *Characidium orientale*: CIFURG 171; *Characidium rachovii*: CIFURG 54, 119, 139, 151, 185; *Charax stenopterus*: CIFURG 41, 98, 130, 158, 181, 187; *Cheirdon ibicuhiensis*: CIFURG 16, 32, 45, 52, 63, 89, 127, 155, 172, 177, 198; *Cheirodon interruptus*: CIFURG 53, 72, 102, 144, 168; *Cichlasoma portoalegrense*: CIFURG 21, 73, 100, 140, 165, 186; *Corydoras paleatus*: CIFURG 164, 188; *Crenicichla lepidota*: CIFURG 33, 91; *Ctenogobius shufeldti*: CIFURG 93, 104; *Cyanocharax alburnus*: CIFURG 44; *Cynopoecilus melanotaenia*: CIFURG 112, 121; *Cyphocharax voga*: CIFURG 13, 27, 43, 60, 71, 81, 92, 129, 135, 154, 163, 176, 195; *Geophagus brasiliensis*: CIFURG 84, 103; *Gymnogeophagus* sp.: CIFURG 46; *Hisonotus laever*: CIFURG 105; *Hoplias malabaricus*: CIFURG 34, 59, 122, 138, 157, 189; *Hoplosternum littorale*: CIFURG 17; *Hyphessobrycon boulengeri*: CIFURG 48, 114, 118; *Hyphessobrycon igneus*: CIFURG 10, 31, 38, 56, 66, 87, 94, 115, 116, 134, 142, 156, 182; *Hyphessobrycon luetkenii*: CIFURG 14, 26, 40, 61, 64, 88, 95, 128, 147, 160,174, 178, 199; *Hyphessobrycon togoi*: CIFURG 106; *Jenynsia multidentata*: CIFURG 75; *Lycengraulis grossidens*: CIFURG 76; *Mugil Liza*: CIFURG 80; *Odontesthes argentinensis*: CIFURG 108; *Oligosarcus jenynsii*: CIFURG 12, 28, 42, 69, 77, 131, 149, 183, 194; *Oligosarcus robustus*: CIFURG 29, 47, 55, 68, 79, 97, 132, 137, 150, 170, 184, 193; *Phalloceros caudimaculatus*: CIFURG 11, 30, 36, 49, 83, 111, 120, 133, 152, 192; *Phalloptychus iheringii*: CIFURG 37; *Pimelodella australis*: CIFURG 18, 190; *Platanichthys platana*: CIFURG 74; *Pseudocorynopoma doriae*: CIFURG 19; *Rhamdia quelen*: CIFURG 20, 86, 136, 166; *Steindachnerina biornata*: CIFURG 161; *Synbranchus marmoratus*: CIFURG 141.
